# Concurrent resistance and aerobic training as protection against heart disease

**Published:** 2010-08

**Authors:** Ina Shaw, Brandon S Shaw, Jones F Cilliers, Gregory A Brown

**Affiliations:** Department of Marketing and Sport Management, Vaal University of Technology, Vanderbijlpark, South Africa; Department of Sport, Rehabilitation and Dental Sciences, Tshwane University of Technology, Pretoria, South Africa; Department of Sport, Rehabilitation and Dental Sciences, Tshwane University of Technology, Pretoria, South Africa; Human Performance Laboratory, HPERLS Department, University of Nebraska, Kearney, USA

**Keywords:** cardiovascular disease, endurance training, exercise, risk assessment, weight training

## Abstract

**Summary:**

This study was designed to compare the effects of aerobic and concurrent aerobic and resistance training on their ability to slow the rate of development and progression of coronary heart disease (CHD) in young adult males at low risk, as determined by the Framingham risk assessment (FRA) score. Subjects were assigned to 16 weeks of three-times weekly aerobic training (AT) (*n* = 13), concurrent aerobic and resistance training (CART) (*n* = 13) or no exercise (NO) (*n* = 12). Both AT and CART resulted in significant (*p* < 0.05) changes in total cholesterol (from 173.67 ± 29.93 to 161.75 ± 26.78 mg.dl^-1^ and from 190.00 ± 38.20 to 164.31 ± 28.73 mg.dl^-1^, respectively), smoking status (from 12.25 ± 5.08 to 10.33 ± 5.37 cigarettes per day and 12.00 ± 4.71 to 8.77 ± 5.10 cigarettes per day, respectively), high-density lipoprotein cholesterol (from 47.00 ± 11.85 to 57.50 ± 5.99 mg.dl^-1^ and 34.00 ± 8.53 to 46.77 ± 14.32 mg.dl^-1^, respectively), systolic blood pressure (from 126.17 ± 7.00 to 122.33 ± 3.17 mmHg and 131.54 ± 9.28 to 121.69 ± 7.87 mmHg, respectively) and therefore FRA score (from 3.58 ± 2.19 to 1.33 ± 2.27 and 5.77 ± 3.09 to 2.46 ± 2.90, respectively). Both modes of exercise were found to be equally effective in reducing CHD risk. These findings support the inclusion of resistance training into an aerobic training programme to lower CHD risk, which will afford subjects the unique benefits of each mode of exercise.

## Summary

Coronary heart disease (CHD) is the leading cause of death in western and developed countries, contributing to 60% of all deaths in the United States of America.^1^,^2^ The Centers for Disease Control and Prevention (CDC) and the American College of Sports Medicine (ACSM) recommend that to prevent CHD, every adult should achieve at least 30 minutes of moderate-intensity physical activity on preferably all days of the week.^3^ While many studies have confirmed the benefit of exercise in reducing the overall risk of CHD,^4^ according to the United States’ Department of Health, three out of five (60%) individuals in the United States do not achieve the required level of exercise, while a further 25% of the adult population do not exercise at all.^5^

The Framingham risk assessment (FRA) score is the most widely accepted tool in the United States for patient selection for primary intervention and prediction of coronary events over the next 10 years.^6^ The National Cholesterol Education Program (NCEP) Adult Treatment Panel (ATP) III guidelines recommend FRA risk scoring in assessing absolute risk for CHD events.^7^

The search for the cause of CHD was started in the 1940s in the small community of Framingham in the United States.^8^ The Framingham Heart study was designed to generate information that would assist in the prevention and early detection of CHD. It is now the source of much of the knowledge on the risk of CHD and is synonymous with the ‘risk factor hypothesis’.

The use of the FRA tool is especially important as it is designed to estimate risk in adults who do not yet have CHD. The FRA tool also determines CHD risk from a group of variables (i.e. age, smoking status, systolic blood pressure, total cholesterol and high-density lipoprotein cholesterol), rather than from a single variable.^9^ This FRA score can then be utilised to identify patients for primary or preventative treatments, including exercise.

Over several decades, volumes of information have been produced on aerobic training, its adaptations and its benefits in preventing CHD.^10^ Although the specific cardioprotective benefits of aerobic training are well known, resistance training is becoming increasingly popular among the general public and individuals who have substantial mobility limitations (especially the elderly), which preclude aerobic methods of exercise. This necessitated the determination of the effects of this mode of training on CHD risk. Research may reveal additional benefits from the inclusion of resistance training with or instead of more traditional modes of aerobic exercise. Well-known benefits of resistance training include increased muscle strength, increased lean tissue mass, maintenance of or increase in metabolically active tissue, and increased neuromuscular control and coordination.^11^

While it is commonly accepted that resistance training should be regarded as a complementary mode of exercise and not a substitute for aerobic training,^11^ unfortunately, it is not well understood what the effect of a concurrent aerobic and resistance training programme would be on CHD risk factors. Just as CHD is not a single condition, no single exercise-training programme is optimal for risk reduction in all individuals.^12^ Furthermore, while the necessary quantity and intensity of exercise for the primary prevention of CHD are becoming understood, most research to date focuses on the effect of a single mode of exercise on a single CHD risk variable in isolation.

The purpose of this study was to compare the effect of aerobic exercise alone, with that of aerobic combined with resistance training on the FRA scores of young males with few CHD risk factors. A finding that a combination of aerobic and resistance training could reduce several CHD risk factors simultaneously would be of critical importance, due to the synergistic benefits to be gained from each mode of exercise.

## Methods

Thirty-seven sedentary young adult males volunteered to participate in this study (mean age 25 years and six months). Demographic data are presented in Table 1. Age was ascertained from date of birth. Since females may have a greater variability in CHD risk factors than males, only males were selected.^13^ The subjects were required to be free of any medical conditions that could have prevented them from optimising the benefits of their exercise training, they had to be inactive and weight stable at least six months before the study started and were not allowed to be on any weight-management programmes or pharmacological agents that could have affected the measured CHD risk factors.

**Table 1.** Baseline Subject Descriptive Data

	*Group*		
*Variables*	*NO (n = 12)*	*AT (n = 12)*	*CART (n = 13)*
Age (years)	25 ± 2.4	25 ± 5.6	26 ± 3.1
Height (cm)	179.3 ± 11.9	176.8 ± 3.8	178.7 ± 7.0
Body mass (kg)	80.3 ± 12.8	74.7 ± 8.2	85.0 ± 12.8

Values are means ± standard deviation

All subjects underwent an identical battery of tests before and after the 16-week intervention period. All subjects were evaluated in the post-absorptive state following a 12- to 14-hour fast and at least 48 hours prior to or following any exercise. Prior to participation in the investigation, all volunteers gave written informed consent and underwent a screening history and physical examination. They were allowed to discontinue the study at any time.

This investigation was approved by the Institutional Review Board at the University of Johannesburg (formerly Rand Afrikaans University). Random assignment was made to one of the experimental exercise groups: aerobic training (AT) (*n* = 12) or concurrent aerobic and resistance training (CART) (*n* = 13), or to the non-exercising (NO) group (*n* = 12), using a schedule generated from a table of random numbers.

For descriptive purposes, anthropometric measurements were carried out according to the methods proposed by the International Society for the Advancement of Kinanthropometry (ISAK).^14^ Subjects were weighed to the nearest 0.1 kg on a calibrated medical scale (Mettler DT Digitol, Mettler-Toledo AG, Ch-8606 GreiFensee, Switzerland) wearing only running shorts. Each subject’s height was measured to the nearest 0.1 cm via a standard wall-mounted stadiometer.

## FRA score calculation

The variables used in FRA calculation to estimate risk in adults who do not yet have CHD are age, smoking status, systolic blood pressure, total cholesterol value and high-density lipoprotein cholesterol value. Information on smoking habits in terms of the number of cigarettes smoked daily was obtained by a self-administered seven-day questionnaire a week before the pre- and post-test.^15^ This was done in order to establish whether any modifications in the subject’s smoking habits took place during the course of the treatment period. At entry into the study, all subjects participated in a half-hour session on how to estimate the number of cigarettes smoked daily and how to complete the smoking form.

Each subject’s resting systolic (SBP) and diastolic blood pressure (DBP) was measured in the supine (recumbent) position after five minutes’ rest using a mercury sphygmomanometer (Alpk2 Sphygmomanometer, Japan). The mercury column was positioned at the same level as the subject’s heart during monitoring. The left arm of each subject was supported and utilised throughout the investigation. Subjects provided blood samples in the sitting position and samples were analysed for total cholesterol and high-density lipoprotein cholesterol concentrations following a nine- to 12-hour fast and prior to any exercise.^16^ Each parameter was assayed on a single day to eliminate inter-assay variability. For estimation of each subject’s FRA score, the present study utilised the point system of the Framingham Heart study.^6^

## Training design

All subjects participated in three 60-minute exercise sessions each week for 16 weeks. The aerobic training sessions started with the subjects warming up by cycling for five minutes at a heart rate of less than 100 beats per minute, followed by rowing, stepping, cycling and walking on a treadmill for a total of 45 minutes at 60% of their individual heart rate maximum. They cooled down with a five-minute cycle at a heart rate of less than 100 beats per minute. Age-predicted maximum heart rates were determined by subtracting their age from 220. Heart rate was measured continuously during the training sessions with a heart rate monitor (Polar Fitwatch, Polar Electro Oy, Finland).

The intensity of exercise was increased by 5% every four weeks.^17^,^18^ In an attempt to equalise for time across the two exercise groups, the subjects in the CART group had to perform 22 minutes of both aerobic training and resistance training.

The resistance training component of the programme required that the subjects perform two sets of 15 repetitions at 60% of one-repetition maximum (1-RM) for each of the eight prescribed exercises, which included shoulder press, latissimus dorsi pulldowns, seated chest press, low pulley row, crunches, unilateral leg press, unilateral knee extensions and unilateral prone hamstring curls. Subjects were under direct supervision during the training sessions, and all subjects were familiarised with the equipment before commencement of the experimental programmes.

The CART subjects had to perform a similar warm-up and cool-down protocol to that of the AT subjects. The weight training intensity was re-evaluated through 10-RM testing and the training programmes were adjusted by increasing the resistance accordingly to maintain 60% of the estimated 1-RM every four weeks.

The subjects in the NO group were instructed to remain inactive during the 16-week period.

## Statistical analyses

Quantitative data are expressed as means and standard deviations (SD). While Levene’s test was used to determine the equality or homogeneity of variance of the groups, the Brown and Forsythe *F*-test was utilised to determine equality of means. Following this, the present investigation also computed dependent *t*-tests to determine whether a significant difference existed between pre- and post-tests. Differences in anthropometric measures were compared using a one-way analysis of variance (ANOVA). When significant differences were found at the post-test, a Dunnett *post hoc* analysis was employed.

The control group’s data was utilised to calculate test–retest reliability by quantifying it using the intra-class correlation coefficient. Statistical significance was indicated by *p* ≤ 0.05. Statistical analysis was performed with the Statistical Package for Social Sciences (SPSS) version 14 (Chicago, IL).

## Results

Mean pre- and post-training values are reflected in Table 2. The NO, AT and CART groups were found to be statistically similar at the start of the study regarding total cholesterol levels (*p* = 0.084), smoking status (*p* = 0.763), high-density lipoprotein cholesterol levels (*p* = 0.201), systolic blood pressure (*p* = 0.339) and FRA scores (*p* = 0.548).

**Table 2.** Changes In Framingham Risk Assessment Scores Following Aerobic And Concurrent Aerobic And Resistance Training In Healthy Previously Sedentary Young Adult Males

*Variables*	*Group*					
	*NO (n = 12)*		*AT (n = 12)*		*CART (n = 13)*	
	*Pre-training*	*Post-training*	*Pre-training*	*Post-training*	*Pre-training*	*Post-training*
TC (mg.dl^-1^)	195.75 ± 10.49	194.83 ± 11.85	173.67 ± 29.93	161.75 ± 26.78*	190.00 ± 38.20	164.31 ± 28.73*
Smoking status (cigarettes per day)	12.42 ± 4.83	12.33 ± 4.77	12.25 ± 5.08	10.33 ± 5.37*	12.00 ± 4.71	8.77 ± 5.10*
HDL-C (mg.dl^-1^)	49.33 ± 6.34	50.17 ± 8.97	47.00 ± 11.85	57.50 ± 5.99*	34.00 ± 8.53	46.77 ± 14.32*
SBP (mmHg)	122.00 ± 5.72	124.67 ± 4.77*	126.17 ± 7.00	122.33 ± 3.17*	131.54 ± 9.28	121.69 ± 7.87*
Total FRA score	5.00 ± 2.80	4.67 ± 2.46	3.58 ± 2.19	1.33 ± 2.27*	5.77 ± 3.09	2.46 ± 2.90*

TC: total cholesterol; HDL-C: high-density lipoprotein cholesterol; SBP: systolic blood pressure; FRA: Framingham risk assessment *Significantly different when compared with pre-training values (*p* ≤ 0.05).

The data indicated that while there was no change in total cholesterol levels in the NO group from pre- to post-training (*p* = 0.678), both the AT and CART groups were found to have significantly (*p* < 0.05) decreased total cholesterol levels at post-training (*p* = 0.004 and *p* = 0.007, respectively). Further post hoc analysis revealed that the aerobic and concurrent training had a comparable effect on total cholesterol levels (*p* = 0.324) and that both were more effective than no exercise (*p* = 0.004 and *p* = 0.008, respectively).

At the end of the 16-week period, there were no significant differences in the number of cigarettes smoked by the three groups. While the high-density lipoprotein cholesterol levels of the NO group did not change (*p* = 0.672), the AT and CART groups had increased high-density lipoprotein cholesterol levels (*p* = 0.001 and *p* = 0.001, respectively). Aerobic training was found to be as effective as concurrent training at increasing high-density lipoprotein cholesterol levels (*p* = 1.000) and both were more effective than no exercise (*p* = 0.030 and *p* = 0.005, respectively). Sixteen weeks of aerobic and concurrent training reduced systolic blood pressure (*p* = 0.049 and *p* = 0.002, respectively). However, the NO group was found to have significantly increased mean systolic blood pressure (*p* = 0.025).

*Post hoc* analysis revealed that aerobic and concurrent training proved equally effective for improving systolic blood pressure levels (*p* = 0.097). However, only concurrent training was more effective than no exercise (*p* = 0.000). FRA scores were not reduced in the NO group (*p* = 0.504), whereas both aerobic and concurrent training proved effective in reducing their FRA scores (*p* = 0.001 and *p* = 0.000). Aerobic and concurrent training were found to be equally effective (*p* = 0.484).

## Discussion

The FRA equation, which makes use of a group of variables rather than a single variable, is an accepted method of estimating absolute risk for CHD and is especially important in estimating the 10-year risk in adults who do not yet have CHD. Although most research to date focuses on a single mode of exercise and how it affects a single CHD risk variable, the present study provides new data on how two modes of exercise training influence the FRA scores of young adult males with few CHD risk factors.

We found that aerobic and concurrent aerobic and resistance training had a favourable effect on levels of total cholesterol and high-density lipoprotein cholesterol, systolic blood pressure and FRA score. This is noteworthy, since concurrent training decreased FRA score by the same amount as aerobic training alone, despite the fact that the subjects who engaged in the concurrent training did only half as much aerobic training as the aerobic-only training group.

With regard to the effect of aerobic training on the individual variables determining the FRA scores, several studies have found that this mode of exercise training had a favourable effect on levels of total cholesterol^19^-^22^ and high-density lipoprotein cholesterol,^4^,^17^,^20^,^23^ and systolic blood pressure.^20^,^24^ Although the effect of aerobic training on the individual FRA variables is well documented,^25^ less is known about the effect of concurrent aerobic and resistance training.^26^ The present investigation obtained a seemingly novel result, with concurrent training resulting in a significant reduction in total cholesterol levels. While this study demonstrated increased high-density lipoprotein cholesterol values following concurrent training, Wallace *et al*.^27^ did not find significantly increased levels following their concurrent training programme. Although their study supports the findings of the present study, that concurrent training reduced systolic blood pressure, the study of Pierson *et al*.^18^ refutes these findings as they found unchanged systolic blood pressure values following concurrent training.

## Conclusion

These findings are noteworthy since the addition of resistance training to aerobic training can now be considered an alternative and effective treatment to reduce the risk and prevalence of CHD in males. Participation in activities that require both aerobic and resistance components should be considered in future as a tool to lower absolute risk for CHD, since each mode of exercise has unique benefits.
